# Supplementary material for: A New Immortalized Human Lacrimal Gland Cell Line
Source: Cells. 2024 Apr 3;13(7):622. doi: 10.3390/cells13070622 (PMC11011892; doi:10.3390/cells13070622)
Supplement: Supplementary file 1 [file cells-13-00622-s001.zip › cells-2898168-supplementary.pdf]

Supplemental material for

A new immortalized human lacrimal gland cell line

Sophie Gleixner *et al.*

*Corresponding author. Email: philipp.arnold@fau.de

This PDF file includes:

Figures S1 to S5
Table S1 and S2

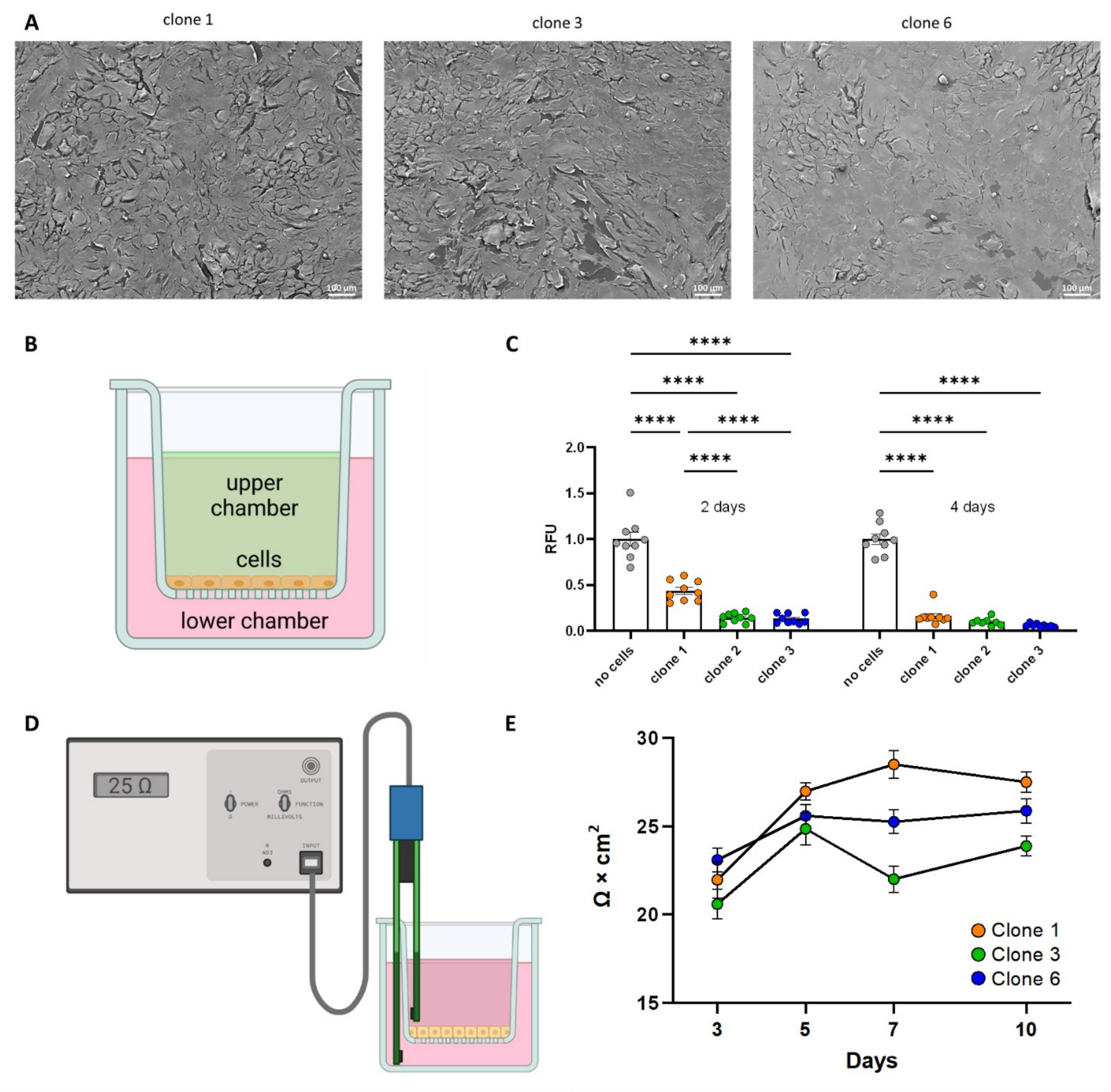

Figure S1.

Validation of the epithelial character of three immortalized human lacrimal gland cell clones. **A**) Scanning electron microscopy of the surface of clone 1, 3 and 6 after cells reached confluence. **B**) Cartoon of FITC-dextran permeability assay setup. The figure is partly created with BioRender.com. **C**) Relative fluorescence units (RFU) measured after 30 minutes in the lower chamber of wells with cell culture inserts containing cells of clone 1, 3 and 6 after 2 or 4 days of growth. Data are means ± SEM [*$p < 0.05$, **$p < 0.01$, ***$p < 0.001$, and ****$p < 0.0001$, Two-way ANOVA, N = 3]. **D**) Cartoon of TEER measurement setup. The figure is partly created with BioRender.com **E**) TEER values measured in transwell inserts containing cells of clone 1, 3 and 6 after 3, 5, 7 and 10 days. Data are means ± SEM [N = 12].

gene	protein
ACTA2	Smooth Muscle Actin Beta 2
AQP5	Aquaporin 5
CD14	Monocyte Differentiation Antigen CD14
CD34	Sialomucine
COL1A2	Collagen Type I Alpha 2 Chain
COL6A2	Collagen Type VI Alpha 2 Chain
CST6	Cystatin 6
CSTB	Cystatin B
DES	Desmin
FN1	Fibronectin 1
FOXC1	Forkhead Box C1
HAS2	Hyaluronan Synthase 2
LUM	Lumican
MFAP5	Microfibril Associated Protein 5
MYL9	Myosin Light Chain 9
PALLD	Palladin
PAX6	Paired Box Gene 6
PDGFRB	Platelet Derived Growth Factor Receptor B[illegible]
POSTN	Periostin
VIM	Vimentin
VWF	Von Willebrand Factor

Table S1.

List of marker genes for lacrimal gland epithelial cells, endothelial cells, mesenchymal stem cells, myoepithelial cells and fibroblasts.

A

gene	protein
CST6	Cystatin 6
FOXC1	Forkhead Box C1
MYL9	Myosin Light Chain 9
PAX6	Paired Box Gene 6
CSTB	Cystatin B
ACTA2	Smooth Muscle Actin Beta 2
C3	Complement C3
KRT5	Keratin 5
TNFRSF19	TNF Receptor Superfamily Member 19
AQP5	Aquaporin 5
CHRM3	Cholinergic Receptor Muscarinic 3
ADRB1	Adrenoceptor Beta 1
HTRA4	HtrA Serine Peptidase 4
MYH11	Myosin Heavy Chain 11
LCN2	Lipocalin 2
VIPR1	Vasoactive Intestinal Peptide Receptor 1
PIGR	Polymeric Immunoglobulin Receptor
SFTPD	Surfactant Protein D
LTF	Lactoferrin
LGR5	Leucine Rich Repeat Containing G Protein-Coupled Receptor 5
SERPINB2	Serpin Family B Member 2

B

gene	protein	size [bp]
ACTA2	Smooth Muscle Actin Beta 2	88
ACTB	Beta Actin	298
AQP5	Aquaporin 5	232
CST6	Cystatin 6	255
CSTB	Cystatin B	243
FOXC1	Forkhead Box C1	82
MYL9	Myosin Light Chain 9	334
PAX6	Paired Box Gene 6	500

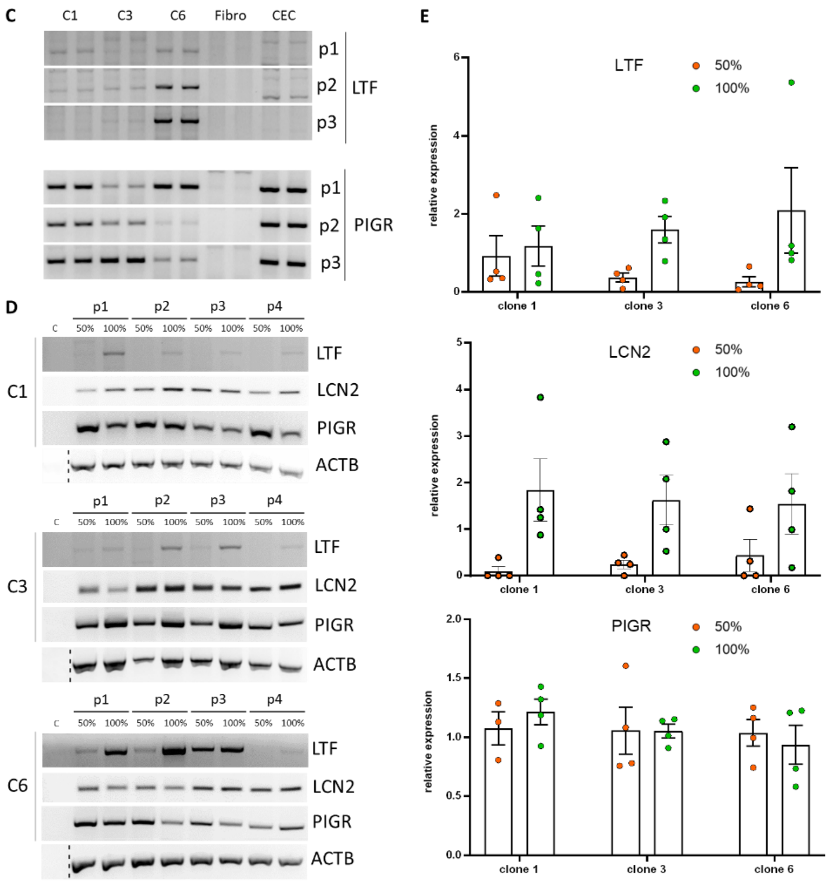

Figure S2.

Comparison of expression of different lacrimal gland marker genes. **A**) List of lacrimal gland marker genes. **B**) List of the marker genes tested in Fig. 3B with gene names, names of the respective protein product and the expected amplicon size. **C**) Agarose gels with the amplification products of RT-PCR for the tear fluid proteins lactoferrin (LTF) and Polymeric Immunoglobulin Receptor (PIGR) at three different passages (p1, p2, p3) for clone 1 (C1), 3 (C3), 6 (C6), human fibroblasts (Fibro) and human corneal epithelial cells (CEC). **D**) Agarose gels with the amplification products of RT-PCR for LTF, LCN2, PIGR and actin beta (ACTB) at 50 % and 100 % confluence at four different passages (p1-p4) for clone 1, 3 and 6 and a negative control (C). **E**) Semiquantitative comparison of LTF, LCN2 and PIGR expression level in clone 1, 3 and 6 at 50 % and 100 % confluence. Data are means ± SEM [Two-way ANOVA, N = 4].

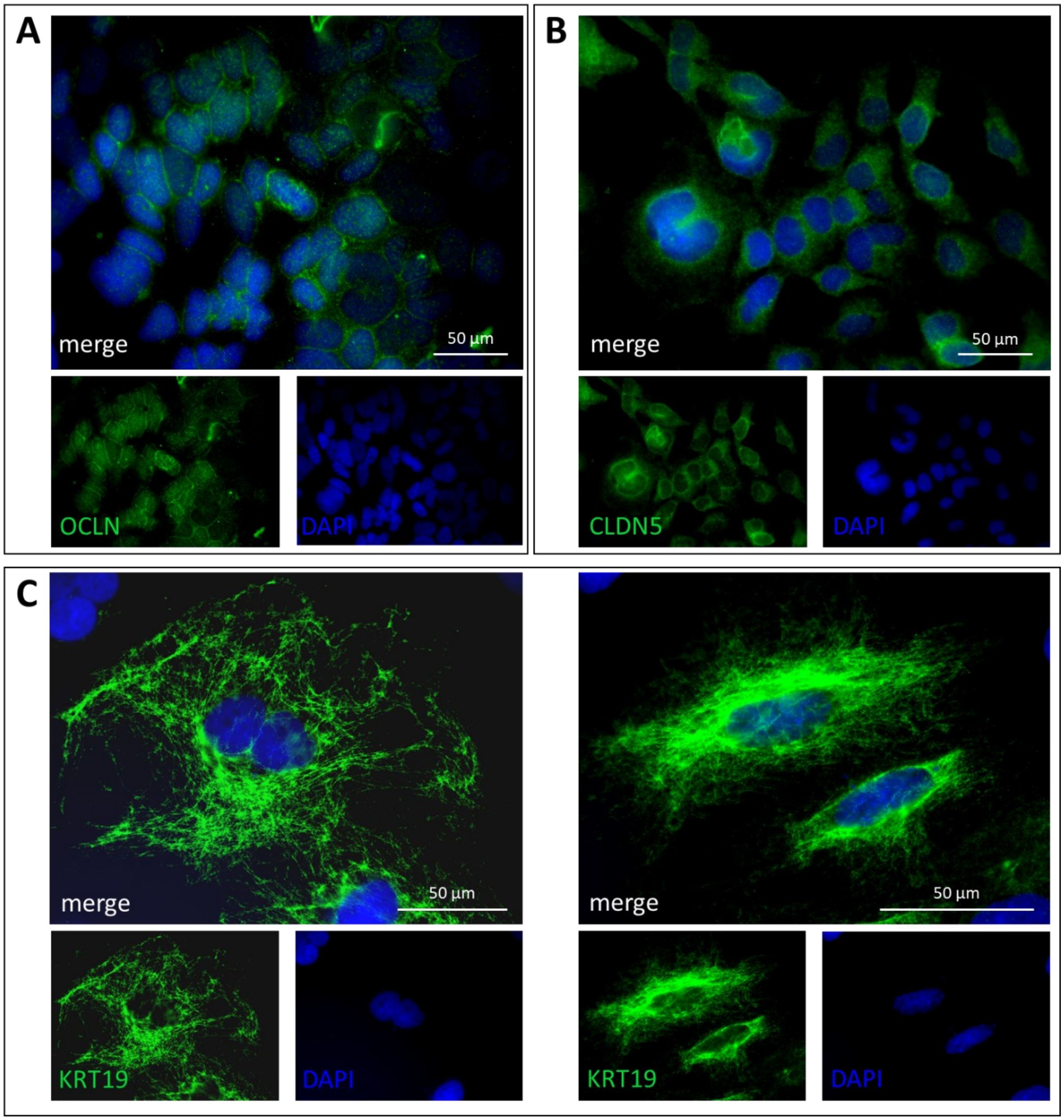

Figure S3.

Immunofluorescence image of clones 1, 3 and 6 stained against DAPI (blue) and occludin (OCLN, green, A, clone 1), claudin 5 (CLDN5, green, B, clone 3) and cytokeratin 19 (KRT19, green, C, clone 1 left, clone 6 right).

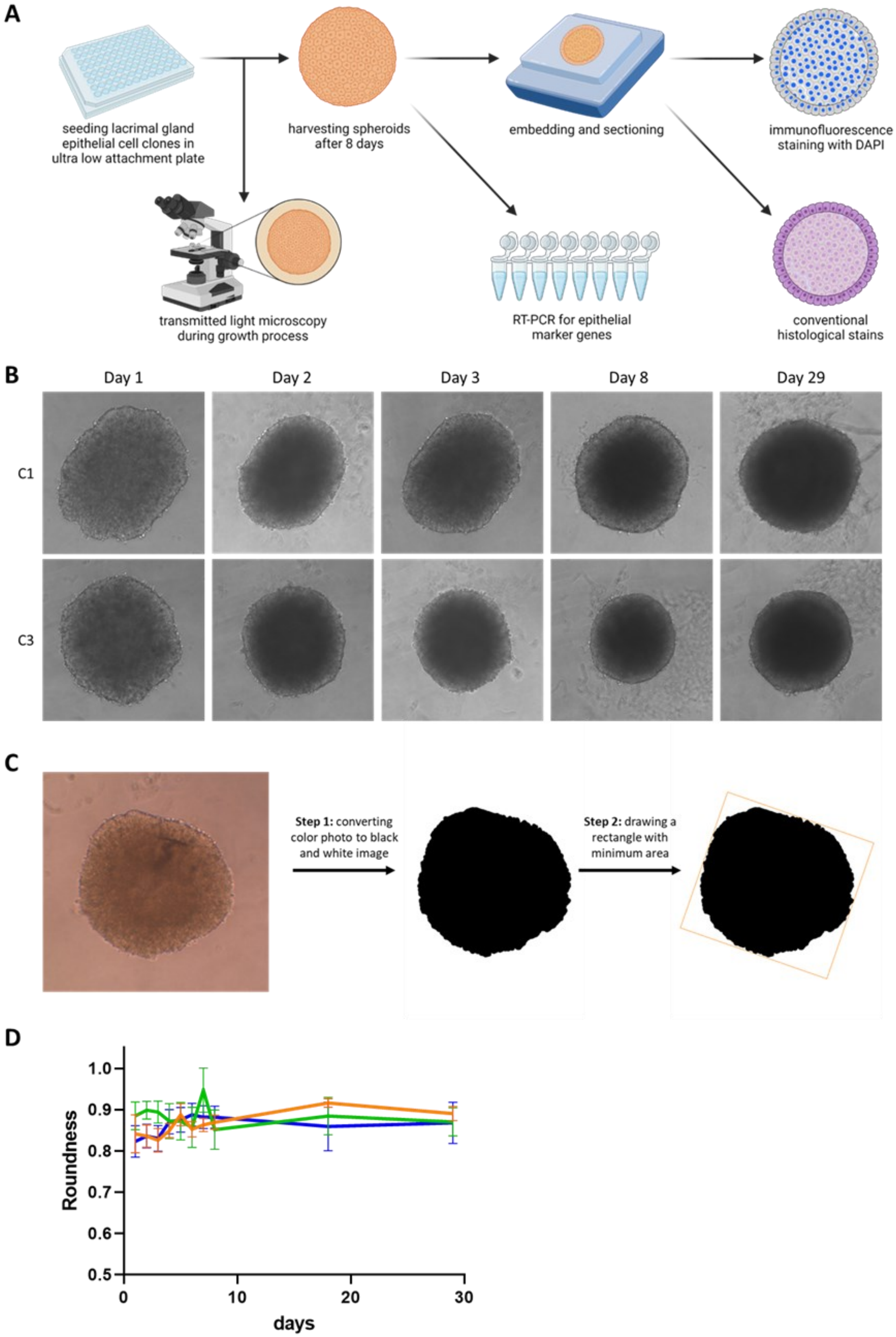
A
seeding lacrimal gland epithelial cell clones in ultra low attachment plate
harvesting spheroids after 8 days
embedding and sectioning
immunofluorescence staining with DAPI
transmitted light microscopy during growth process
RT-PCR for epithelial marker genes
conventional histological stains
B
Day 1
Day 2
Day 3
Day 8
Day 29
C1
C3
C
Step 1: converting color photo to black and white image
Step 2: drawing a rectangle with minimum area
D
Roundness
1.0
0.9
0.8
0.7
0.6
0.5
0
10
20
30
days

Figure S4.

Analysis of spheroidal growth. **A**) Cartoon illustrating the workflow from the lacrimal gland cells clones to 3D spheroids and their analysis. The figure was partly created with BioRender.com. **B**) Transmitted light microscopy during the 29 days culture period of 3D grown cells of clone 1 (C1) and 3 (C3). **C**) To determine the circularity of the spheroids, in the first step the microscopic images were converted to black and white images for representing each spheroid as a black object on white background. In the second step, these images were analyzed using an in-house Python script for measuring the size of objects in an image with OpenCV. This script draws a rectangle with the minimum area around the black object and calculates the edge lengths of the rectangle. For exactly circular objects, both edges of the rectangle have the same length, while for elliptical objects the edge lengths differ. **D)** Roundness of the spheroids of clone 1 (orange), 3 (green) and 6 (blue) over the course of the 29 days culture period. Data are means ± SEM. [Day 1-8 N = 24, day 18 N= 9, and 29 N = 9].

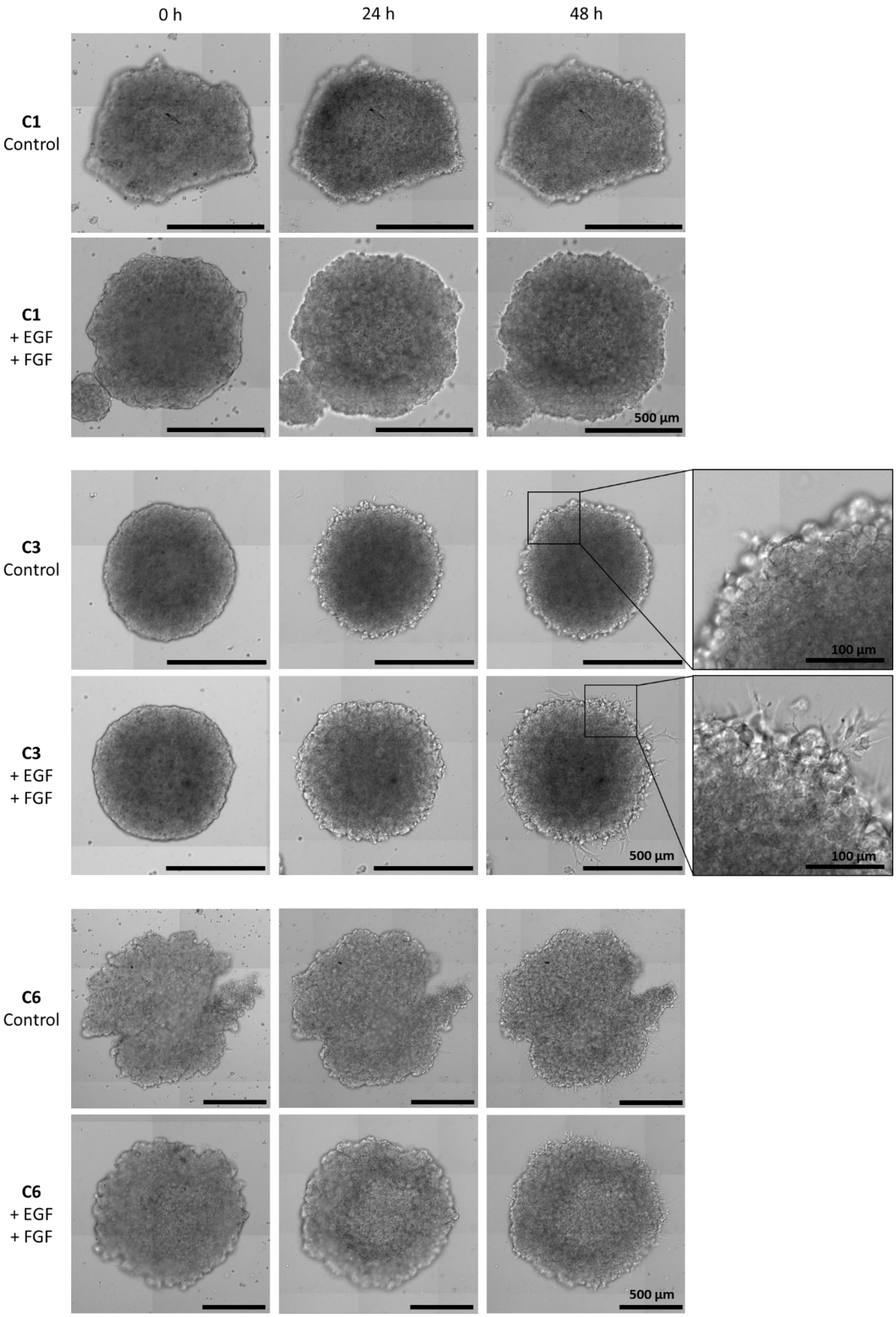

0 h
24 h
48 h
C1
Control
C1
+ EGF
+ FGF
500 µm
C3
Control
100 µm
C3
+ EGF
+ FGF
500 µm
100 µm
C6
Control
C6
+ EGF
+ FGF
500 µm

Figure S5.

Spheroids in extracellular matrix with and without growth factors. Epidermal growth factor (EGF) and fibroblast growth factor 10 (FGF10) added to the medium form buds after 0, 24 and 48 hours.

Abbreviation	Gene name
ACTA2	Actin Alpha 2, Smooth Muscle
ACTB	Actin beta
ADRB1	Adrenoceptor Beta 1
AQP5	Aquaporin 5
ATP5G3	ATP Synthase Membrane Subunit G Locus 3
C3	Complement C3
CADH1	E-cadherin
CCL28	C-C Motif Chemokine Ligand 28
CD14	Monocyte Differentiation Antigen CD14
CD34	Sialomucine
CD90, THY1	Thy-1 Cell Surface Antigen
CHRM3	Cholinergic Receptor Muscarinic 3
Col1A2	Collagen Type I Alpha 2 Chain
Col6A2	Collagen Type VI Alpha 2 Chain
CST6	Cystatin 6
CSTB	Cystatin B
DCBLD2	Discoidin, CUB And LCCL Domain Containing 2
DES	Desmin
EEF1A1	Eukaryotic translation elongation factor 1 alpha
EHF	ETS Homologous Factor
FN1	Fibronectin 1
FOXC1	Forkhead Box protein C1
HAS2	Hyaluronan Synthase 2
HSPG2	Heparan Sulfate Proteoglycan 2
HTRA4	HtrA Serine Peptidase 4
Hu18s, 18S	18S ribosomal RNA
LCN2	Lipocalin 2
LGR5	Leucine Rich Repeat Containing G Protein-Coupled Receptor 5
LMO4	LIM Domain Only 4
LTF	Lactotransferrin
LUM	Lumican
LYZ	Lysozyme
MFAP5	Microfibril Associated Protein 5
MT2A	Metallothionein 2A
MYH11	Myosin Heavy Chain 11

MYL9	Myosin Light Chain 9
PALLD	Palladin
PAX6	Paired Box Gene 6
PDGFRB	Platelet Derived Growth Factor Receptor Beta
PIGR	Polymeric immunoglobulin receptor
POSTN	Periostin
PXDN	Peroxidasin
RBM47	RNA Binding Motif Protein 47
RPL10	Ribosomal protein 10
RPL21	Ribosomal protein 21
RPL3	Ribosomal protein 3
RPL7	Ribosomal protein 7
SERPINB2	Serpin Family B Member 2
SFTPD	Surfactant Protein D
SKA2	Spindle And Kinetochore Associated Complex Subunit 2
SLC14A1	Solute Carrier Family 14 Member 1 (Kidd Blood Group)
SMC2	Structural maintenance of chromosomes 2
SMC4	Structural maintenance of chromosomes 4
SRPRA	SRP receptor subunit alpha
STK39	Serine/Threonine Kinase 39
STMN1	Stathmin 1
SV40	Simian virus antigen 40
TNFRSF19	TNF Receptor Superfamily Member 19
TNKS1BP1	Tankyrase 1 Binding Protein 1
TOP2A	DNA topoisomerase II alpha
TRIM2	Tripartite Motif Containing 2
VIM	Vimentin
VIPR1	Vasoactive Intestinal Peptide Receptor 1
VWF	Von Willebrand Factor

Table S2.

List of abbreviations and gene names.
